# A refinement to eRNA and eDNA-based detection methods for reliable and cost-efficient screening of pathogens in Atlantic salmon aquaculture

**DOI:** 10.1371/journal.pone.0312337

**Published:** 2024-10-21

**Authors:** Ottavia Benedicenti, Marit Måsøy Amundsen, Saima Nasrin Mohammad, Trude Vrålstad, David A. Strand, Simon Chioma Weli, Sonal Patel, Hilde Sindre

**Affiliations:** 1 Norwegian Veterinary Institute, Ås, Norway; 2 Norwegian Veterinary Institute, Bergen, Norway; University of Hyogo, JAPAN

## Abstract

Finfish aquaculture is one of the fastest-growing food production sectors in the world, and numerous infectious diseases are a constant challenge to the fish farming industry, causing decreased fish health and, consequently, economic losses. Specific and sensitive tools for pathogen detection are crucial for the surveillance of environmental samples to prevent the spread of fish pathogens in farms. Monitoring of waterborne pathogens through filtration of water and subsequent molecular detection of target-specific DNA or RNA sequence motifs is an animal-friendly method. This approach could reduce or even replace the sacrifice of fish for monitoring purposes in aquaculture and allow earlier implementation of disease control measures. Sampling methods might be a bottleneck, and there is a need for simple sampling methods that still ensure the best detection probability. In this study, we tested different filtration methods with spiked freshwater and seawater for a panel of fish pathogens to discern a suitable procedure that can be easily applied on-site by farm personnel without compromising detection probability. Specifically, we tested combinations of different filtration flow rates, lysis buffers, and filters for the detection of some of the pathogens relevant to the aquaculture industry. The results showed that a “sandwich” filtration method using two different filters and a flow rate of up to 4.0 L/min ensured good pathogen detection. The filters, consisting of a hydrophilic glass fibre filter with binder resin on the top and a hydrophilic mixed cellulose esters membrane at the bottom, achieved the best concentration and qPCR detection of both viral and bacterial fish pathogens. This up-and-coming tool allows the detection of very different fish pathogens during a single filtration step, and it can be combined with one single automated total nucleic acid extraction step for all the investigated pathogens, reducing both analysis costs and time.

## Introduction

Finfish aquaculture is one of the world’s fastest-growing food production sectors, with over 50 million tonnes produced annually in recent years [1]. In 2020, Atlantic salmon, *Salmo salar* (Linnaeus), production reached 32.6% of the total finfish marine and coastal aquaculture, being currently a highly accessible and affordable source of animal proteins and playing a vital role in the food and nutrition security of growing human populations [1]. However, numerous infectious diseases are a constant challenge to the salmon farming industry, causing impaired fish health, huge welfare issues, and, consequently, economic losses [2]. To be able to limit the negative effects of the diseases, targeted preventive measures to limit the spread of pathogens in fish farms are necessary. To achieve this, specific and sensitive tools for early pathogen detection are crucial. In the aquaculture sector, current laboratory methods used for diagnosis of salmon diseases are still largely dependent on fish sampling for nucleic acid isolation and amplification, bacteriology, histopathology, and immunohistochemistry (IHC) analyses [2]. Detection of pathogens at early emergence, when the prevalence is low, is difficult with the above-mentioned strategies as salmon farms have high fish densities in the cages, and only a small number of fish can be tested for practical reasons. Moreover, these methods rely heavily on preceding observed cases of disease within the fish population, which will hamper the possibility of early detection and preventive measures of many infectious diseases to control their spread and reduce the on-site consequences of disease.

In recent years, the collection of water samples followed by concentration of dispersed biological material, so-called environmental DNA/RNA (eDNA/eRNA) sampling, has been used increasingly for environmental surveillance and epidemiological studies [3–16]. This method has been a recommended strategy, for instance, after the SARS-CoV-2 pandemic emerged in 2020, as a proxy for early warning of potential COVID-19 outbreak, or as a wastewater-based epidemiology surveillance tool for potential virus transmission via contaminated water [11]. In salmon farms, water sampling can be performed from all cages, and this allows for a good screening of the entire site if the method for sampling, filtration, and detection is practical and sensitive enough to detect pathogens with high specificity even at low concentrations. Moreover, a good screening method could not only be used to detect pathogens shed from infected fish but also to find pathogens circulating in tubes and environmental reservoirs on the farm. This could allow the personnel to take preventive measures earlier when the shedding pathogens are present in the farm environment before clinical symptoms appear in the fish population. For this reason, detecting fish pathogens in the water surrounding farmed fish populations (tanks or cages) would be more cost-effective than sampling fish and potentially yield data on pathogen presence earlier in the course of the infection. Further, this method is non-invasive, animal welfare friendly and may spare fish lives used for monitoring purposes–thus aligning with a 3R (Replacement, Reduction, Refinement) strategy [17].

Some of the current published works on fish and shellfish pathogen screening in water focus on the development and evaluation of methods for concentration and detection of single pathogens, using either eRNA or eDNA, from freshwater or seawater environments [8, 12, 14, 18–23]. For instance, some studies focused on the concentration and detection of viruses affecting farmed fish such as salmonid alphavirus (SAV) [8, 18–21, 23] and infectious salmon anaemia virus (ISAV) [22]. Therefore, this study aimed to develop a protocol that allows for the filtration of different aquaculture pathogens (picking up both eDNA and eRNA) simultaneously for surveillance purposes both in freshwater and seawater facilities. The selection of pathogens used in this study was chosen based on relevance to Norwegian aquaculture, covering different virus types both single-stranded (salmonid alphavirus—SAV, infectious salmon anaemia virus—ISAV) and double-stranded (piscine orthoreovirus—PRV) RNA viruses, a double-stranded DNA virus (salmon gill poxvirus—SGPV) and the bacterium *Yersinia ruckeri*. SAV is the causative agent of pancreas disease (PD) in farmed Atlantic salmon and rainbow trout, *Oncorhynchus mykiss* (Walbaum) [24–26], and it is usually spread horizontally through the water. Infection with SAV and ISAV are notifiable diseases in Norway and affect salmonids in fresh and seawater in multiple countries in Europe [2, 21]. However, in Norway, SAV has been only detected at seawater farm sites [21]. Ongoing surveillance for SAV and ISAV are both regulated through government legislation and voluntary sampling from the fish farming industry. Presently, sampling of fish is the only approved method for screening and sampling costs are substantial. PRV, causing heart and skeletal muscle inflammation (HSMI) in farmed Atlantic salmon [27, 28], and SGPV disease, causing acute respiratory distress and mortality in Atlantic salmon [29–35], are not notifiable but serious diseases challenging fish health and welfare in salmon farming. The gram-negative bacterium *Y*. *ruckeri* causing yersiniosis, also known as enteric redmouth (ERM) [36–39], manifests in Atlantic salmon both before and after sea transfer, but the infection is primarily transmitted during the freshwater phase [2]. A single *Y*. *ruckeri* serotype O1 clone (CC 1) has been responsible for nearly all major yersiniosis outbreaks since the mid-90s [2, 40]. Although the disease is not notifiable, ERM is currently a major concern to the Norwegian aquaculture industry [40].

While eDNA sampling for freshwater pathogens such as *A*. *astaci* and *G*. *salaris* rely on water filtration and filter preservation in the field [4, 7, 9, 12–14], other published methods for detecting viral fish pathogens SAV and ISAV in water rely on water sampling in the field and water filtration in the laboratory [8, 21–23]. These previously published methods on SAV and ISAV require steps of manual handling and equipment sterilization making the procedure time-consuming and unsuitable for filtration in the field. With these time-consuming methods, water storage and transport increase the risk for reduced pathogen recovery due to eDNA/eRNA degradation depending on transport and storage temperature and time before nucleic acid extraction. In this study, we aimed to test different easy-to-use protocols to detect five fish pathogens using both a single filtration and a nucleic acid extraction method. Therefore, different tests were performed for spiked freshwater (tap water) and artificial seawater (ca. 35 ppt) with Atlantic salmon pathogenic RNA viruses (ISAV, SAV genotype 3 and PRV genotype 1), DNA virus (SGPV) and a gram-negative bacterium (*Y*. *ruckeri*) to compare and explore the possibility of using: 1) high-flow rates for reducing the time of filtration; 2) a robust lysis buffer to preserve the pathogens in the field with an ambient storage temperature 3) a putative filter (or set of filters) that allow the filtration of enough water volume to detect different pathogens (both DNA and viral nucleic acids); and 4) automated extraction systems to extract nucleic acids from up to 96 samples in one run for subsequent quantification with qPCR assays.

## Material and methods

### Pathogen culture and isolates

The ISAV isolate used for this study is the Norwegian ISAV isolate Glesvaer/2/90 [41] (NCBI GenBank accession numbers HQ259671.1-HQ259678.1). ISAV was propagated in the Atlantic salmon kidney (ASK) cell line (ATCC® CRL-2747™) as described previously [41, 42] with slight modifications. Briefly, cells were grown at 20°C in T-150 culture flasks containing Leibowitz L-15 medium (Sigma-Aldrich, USA, provided by Merck Life Science AS, Norway) supplemented with 10% foetal bovine serum (FBS), L-Glutamine 2mM (Sigma-Aldrich, USA, provided by Merck Life Science AS, Norway) and Penicillin/Streptomycin/Amphotericin B 100x (Biowest L0010-100, France, provided by VWR, Norway). The virus was inoculated in the cell culture and incubated for approximately seven days at 15°C. Cell culture supernatant was harvested and clarified by centrifugation at 2,000 x g for 10 min at 4°C. The clarified supernatant in 1 mL aliquots was frozen at -80°C. Immunofluorescence for identification of ISAV-infected cells was performed in a 10-fold serial dilution as described previously [43]. Virus titre was calculated according to the 50% end-point method of Reed and Muench [44] and expressed as the dilution of the virus causing 50% tissue infection per millilitre (TCID50/mL). Initial stock concentrations of 5 × 10^5^ TCID50/mL or 1 × 10^7^ TCID50/mL were used for spiking freshwater and artificial seawater, respectively.

The SAV genotype 3 (SAV3) used for this study is a field isolate originating from an outbreak of PD in Atlantic salmon from the Hordaland region of Norway (Isolate 4) [45]. The Chum salmon heart (CHH-1) cell line (ATCC CRL-1680) derived from a Chum salmon (*Oncorhynchus keta*, Walbaum) juvenile normal heart tissue was used in the propagation of SAV, as described previously [46] with slight modifications. Briefly, cells were grown at 20°C in T-150 culture flasks containing Leibowitz L-15 medium (Sigma-Aldrich, USA, provided by Merck Life Science AS, Norway) supplemented with 5% FBS, L-Glutamine 2mM (Sigma-Aldrich, USA, provided by Merck Life Science AS, Norway) and Penicillin/Streptomycin/Amphotericin B 100x (Biowest L0010-100, France, provided by VWR, Norway). SAV3 was used to inoculate the cell culture, and, after seven-day incubation at 15°C, the culture supernatants were harvested, centrifuged, and 1 mL aliquots were stored at -80°C. As for ISAV, immunofluorescence identification of SAV3-infected cells was performed on CHH-1 cells in 10-fold series dilutions. The initial stock concentration of 1 × 10^8^ TCID50/mL was used for spiking artificial seawater.

The PRV genotype 1 (PRV1) is derived from a previous infection trial conducted in 2016 [47] where infected erythrocytes were stored at -80°C as it is not possible to culture. Briefly, three parallel tubes with PRV1-infected pelleted erythrocytes were thawed on ice and 350 μL from each tube were mixed in a joint tube. The mixed erythrocytes were firstly pipetted with a syringe with the needle 10 times to lyse all cells and release the virus, and then centrifuged at 12,000 x g for 10 min at 5°C and the lysates pipetted into a fresh tube (pellet erythrocytes Cq ≈ 19.56).

The material used for the SGPV spiking freshwater experiment came from a previous mild SGPVD outbreak in western Norway where whole fish were stored at -20°C as it is not possible to culture. Before the experiment, gills from one fish (gill tissue Cq ≈ 31.98) were put into three Safe-Lock tubes filled with 1 mL freshwater. Furthermore, the gills were homogenized using 5 mm steel beads with TissueLyser II (Qiagen) at 24 Hz for 5 min two times and mixed into a joint tube before the experiment.

*Y*. *ruckeri* strain (from the Norwegian Veterinary Institute (NVI)– 10705 [48, 49]) was prepared from cryopreserved stocks held at -80°C in Tryptic Soy Broth (TSB) with 20% glycerol and cultured on 5% bovine blood agar (BA) at 22°C as previously described [49]. The absorbance was measured before starting the experiment by adding a full loop from BA culture to 1 mL sterile water with a Shimadzu UV-1800 UV/Visible Scanning Spectrophotometer (OD_600_ = 2.258 in 1 mL; concentration of use with a 1:25 dilution corresponds to a Cq ≈ 16.36; taking into account that OD_600_ of 1 is 2 x 10^9^ CFU/mL [50]).

### Experiments

**Fig 1:**
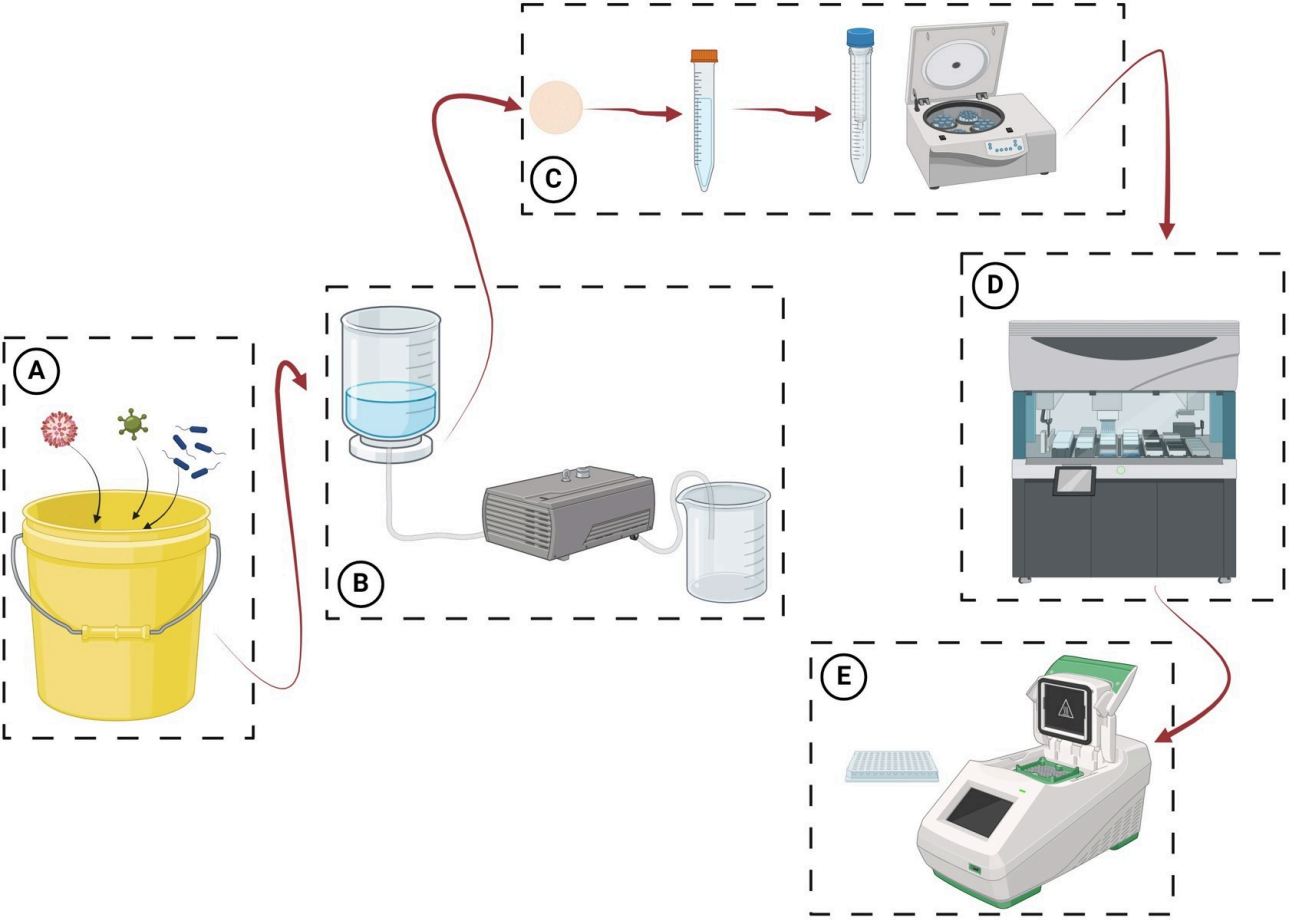
Schematic diagram of the pathogen filtration for detection of environmental nucleic acids. A) Different pathogens in water can be filtered with B) a Nalgene™ Single Use Analytical Filter Funnels (Thermo Fisher Scientific, USA) using different filters and pumps. C) Filters are then stored in lysis buffers until processing. The lysis buffer and the filter are then transferred into a NucleoSpin Filters Midi column (Macherey-Nagel, Germany) and centrifuged at room temperature for 2 min at 5,580 x g to separate the filter from the buffer containing the nucleic acids. D) An automated extraction system such as the MagNA Pure 96 (Roche, France) can be used to extract nucleic acids from the lysis buffer, and ultimately E) from the elution can be performed qPCRs for pathogen detection and quantification. Created with BioRender.com.

In this study, we performed under *in vitro* controlled conditions three experiments aimed to identify the impact of various filtration factors (flow rate and filter type) and storage/extraction buffers on the eDNA/eRNA detection success. Experiment 1 was designed to improve previously published methods [8, 21–23], therefore not all combinations of flow rates, lysis buffers and filters were applied. Experiment 2 was based on the results of experiment 1 and aimed to refine a “sandwich” filtration combining the advantages of two different filter types and tested a temperature-independent storage/lysis buffer practical for sampling in the absence of cooling facilities. Both experiments were conducted in freshwater. Experiment 3 aimed to test the same factors in a seawater setup but based on the freshwater experiments’ results. All experiments have been summarized in a schematic workflow below (Fig 1).

#### Experiment 1 –Freshwater bucket experiment

**Fig 2:**
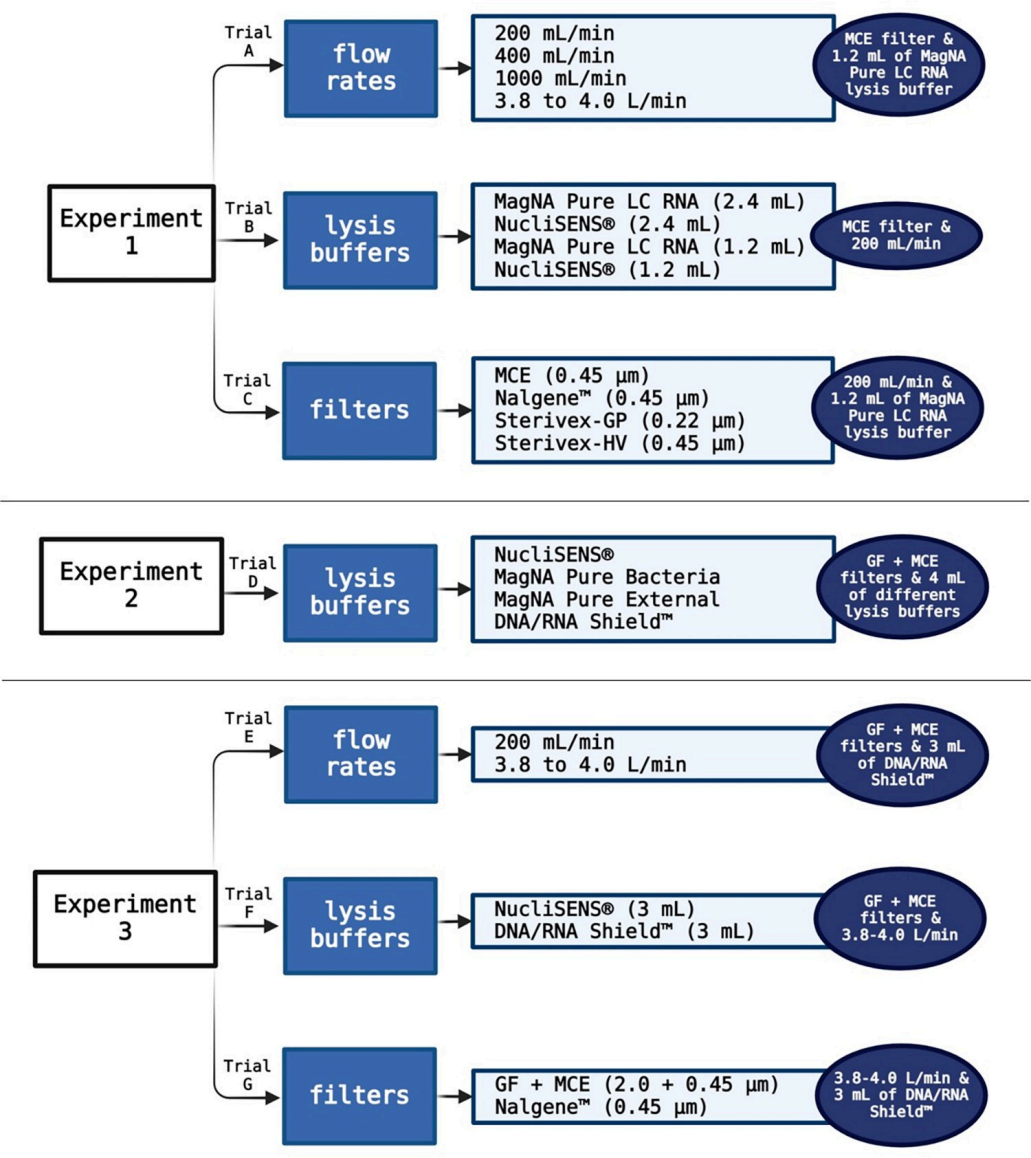
Design of experiments 1, 2 and 3. Experiment 1. Trial A: flow rates were tested with an MCE filter and 1.2 mL of MagNA Pure LC RNA lysis buffer. Trial B: lysis buffers were tested with an MCE filter and a flow rate setting of 200 mL/min (peristaltic pump). Trial C: filters were tested with a flow rate setting of 200 mL/min (peristaltic pump) and 1.2 mL of MagNA Pure LC RNA lysis buffer. Experiment 2. Trial D: four lysis buffers were tested with a “sandwich” filtration method with an MCE filter on the bottom and a GF filter on the top in a Nalgene™ Single Use Analytical Filter Funnels and a flow rate setting of 200 mL/min (peristaltic pump). Experiment 3. Trial E: two flow rates were tested with 3 mL of DNA/RNA Shield™ lysis buffer and the “sandwich” filtration method. Trial F: two lysis buffers were tested with a flow rate setting of 3.8 to 4.0 L/min and the “sandwich” filtration method. Trial G: filters were tested with 3 mL of DNA/RNA Shield™ and 3.8 to 4.0 L/min flow rate setting. Created with BioRender.com.

Experiment 1, based on previously published methods [8, 21–23], tested: 1) different filtration flow rates (trial A); 2) different lysis buffers for filter elution (trial B) and 3) different filters (trial C) (Fig 2 and S1 Table). Each of the parameters in the trials was performed in triplicates (n = 3). For experiment 1, 50 L of tap water were spiked with ISAV (final concentration of 100 TCID50/mL in 50 L, Cq ≈ 29.79 in 1 mL), SGPV (gills from one fish with Cq ≈ 31.98 in 1 mL freshwater added to 50 L), PRV1 (900 μL of pellet erythrocytes with Cq ≈ 19.56 added to 50 L) and *Y*. *ruckeri* (OD_600_ = 2.258 in 1 mL added to 50 L, Cq ≈ 15 in 1 mL) and filtration of 500 mL per filter sample. High initial Cq values for ISAV and SGPV were chosen as we specifically aimed to have an initial pathogen amount comparable to what was found in the tanks during natural infection in the field in previous studies [22]. A tap water sample before pathogens’ spiking was also sampled as negative control and added in all following steps from extraction to amplification. The tap water in the study came from a natural free water source like the typical inlet water for freshwater Atlantic salmon farms and it was treated similarly regarding filtration/UV to remove particles and pathogens. The water was also treated with a low amount of chlorine, typically only 0.3 mg/L water (Ås municipality, Norway, personal communication). For trial A, four different filtration flow rates were tested: 200, 400 and 1000 mL/min flow rate setting with a peristaltic pump (Shenchen, V6-3L, Drifton A/S, Denmark) and a flow rate setting of 3.8 to 4.0 L/min with the EZ-Stream vacuum pump (Millipore, USA). The filter and buffer combination for trial A were the Mixed Cellulose Ester membrane filter (MCE—MF-Millipore® Membrane Filters, 0.45 μm pore size, 47 mm diameter, hydrophilic, Millipore, USA, provided by Merck Life Science AS, Norway) and 1.2 mL of MagNA Pure LC RNA Isolation Tissue lysis buffer (Roche, France).

For trial B, two types of lysis buffers for the preservation of the pathogens were tested: the MagNA Pure LC RNA lysis buffer and the NucliSENS® Lysis Buffer (bioMérieux SA, France). Each lysis buffer was tested for two volumes (1.2 mL and 2.4 mL) using the MCE membrane filter and a flow rate setting of 200 mL/min (peristaltic pump). The chosen buffers are compatible with the automated MagNA Pure 96 nucleic acid extraction protocols (Roche, France). The first lysis buffer is specific for RNA isolation with the MagNA Pure 96 system and the second one has previously been used for preservation and lysis of ISAV and SAV3 [22, 23].

In trial C, four different filters were tested: 1) the MCE membrane filter of 0.45 μm pore size; 2) the cellulose nitrate filter of 0.45 μm pore size, 47 mm diameter, from the Nalgene™ Single Use Analytical Filter Funnels (Thermo Fisher Scientific, USA); 3) the Sterivex-GP Pressure Filter Unit of 0.22 μm pore size, polyethersulfone (PES) membrane (Millipore, USA, provided by Merck Life Science AS, Norway); and 4) the Sterivex-HV Pressure Filter Unit of 0.45 μm pore size, polyvinylidene difluoride (PVDF) membrane (Millipore, USA, provided by Merck Life Science AS, Norway). Each filter was tested with a flow rate of 200 mL/min (peristaltic pump) and 1.2 mL of MagNA Pure LC RNA lysis buffer.

#### Experiment 2 –Freshwater bucket experiment with “sandwich” pathogen filtration

A second freshwater bucket experiment was set up to explore a concept that would allow the filtration of larger water volumes without compromising RNA/DNA yield, and additionally test a wider range of lysis buffers that allow storage at room temperature. Here, we only included ISAV (final concentration of 100 TCID50/mL in 50 L, Cq ≈ 29.79 in 1 mL), PRV1 (900 μL of pellet erythrocytes with Cq ≈ 19.56 added to 50 L) and *Y*. *ruckeri* (OD600 = 2.258 in 1 mL added to 50 L, Cq ≈ 15 in 1 mL). The same amount of tap water was used as for experiment 1 (50 L) but 750 mL (instead of 500 mL) were filtered for each combination of filter and lysis buffer tested. Based on the results from the freshwater bucket experiment 1 analysis, we used a combination of the MCE and Glass Fibre Filter (GF—2.0 μm pore size, hydrophilic glass fibre with a binder resin, and 47 mm diameter, Millipore, USA, provided by Merck Life Science AS, Norway), which is used for large water volume filtration and eukaryotic eDNA capture [12, 13]. We designed a “sandwich” filtration method that aimed to capture the increasingly finer particulates at each stage providing a filtration of all sizes of suspended solids based on results from previous studies [8, 9, 21–23, 51]. Briefly, the Nalgene™ Single Use Analytical Filter Funnels were used for the water filtration and its original cellulose nitrate filter was removed and replaced with a “sandwich” of two filters with the above tested MCE filter (0.45 μm) on the bottom and the GF filter (2.0 μm) on the top (Fig 2 and S1 Table). In addition, we tested four different lysis buffers with the “sandwich” filtration method using a 200 mL/min flow rate setting and 4 mL of each lysis buffer (trial D) in triplicates (n = 3). The chosen amount of lysis buffer per tube was based on previous water filtration with a GF filter [4, 7, 12–14]. The tested buffers were: 1) the NucliSENS® lysis buffer, 2) the MagNA Pure 96 Bacteria lysis buffer (Roche, France), 3) the MagNA Pure 96 External lysis buffer (Roche, France), and 4) the DNA/RNA Shield™ (Zymo Research, USA, provided by Nordic Biosite AS, Sweden).

#### Experiment 3 –Seawater bucket experiment with “sandwich” pathogen filtration

In experiment 3, a seawater experiment, we aimed to test the most promising combinations of filters, buffers and flow rates based on results from the previous freshwater experiments. Here, the “sandwich” filtration method was tested with two flow rates (trial E), two lysis buffers (trial F), and finally a filter control test (trial G) (Fig 2 and S1 Table). Artificial seawater (VWR Chemicals, Norway, 25 L) was spiked with ISAV (final concentration of 800 TCID50/mL in 25 L, Cq ≈ 23.24 in 1 mL), SAV3 (final concentration of 800 TCID50/mL in 25 L, Cq ≈ 21.52 in 1 mL), PRV1 (450 μL of pellet erythrocytes with Cq ≈ 19.56 added to 25 L) and *Y*. *ruckeri* (OD_600_ = 2.258 in 1 mL added to 25 L, Cq ≈ 15 in 1 mL). In each trial, 650 mL were filtered per filter sample. In trial E, two different flow rates (200 mL/min setting with peristaltic pump and 3.8 to 4.0 L/min setting with vacuum pump) were tested with the “sandwich” filtration method described in trial D. The filters were separated and transferred to tubes with 3 mL of DNA/RNA Shield™ buffer. We used 3 mL of DNA/RNA Shield™ as parallel tests in the field showed no significant changes in the pathogen quantification with qPCR assays with a reduction from 4 to 3 mL buffer volume (data not shown). In trial F, 3 mL of the two lysis buffers NucliSENS® and DNA/RNA Shield™ (described in trial D) were tested using the “sandwich” filtration method with a 3.8 to 4.0 L/min flow rate setting. Trial G was performed as a filter method control trial, comparing the “sandwich” filtration method with the filtering procedure using the original cellulose nitrate filter (0.45 μm pore size) from the Nalgene™ Single Use Analytical Filter Funnels. In trial G, a 3.8 to 4.0 L/min flow rate setting was used, and the filters were transferred to 3 mL of DNA/RNA Shield™ lysis buffer.

### gBlocks™ analyses

Sequence-verified gene fragments, or gBlocks™, were ordered from Integrated DNA Technologies (IDT), BVBA (Leuven, Belgium). The gBlocks were designed to cover the ISAV, PRV1, SAV, SGPV and *Y*. *ruckeri* gene fragments (S1 Fig) and estimate the copy numbers. In this study, we ended up not utilizing this system for PRV1 and SGPV.

The gBlocks^TM^ were resuspended in water at 8.4 ng/μL concentration. The copy number/μL in each gBlock^TM^ was calculated by converting the concentration from ng/μL to copy number/μL by using the formula provided by IDT guidelines. After optimisation, the Stock^−3^ dilution (6.25 x 10^4^ copies number/μL) was used as a standard template to estimate the RNA/DNA copy numbers/μL for the cultivable viral (ISAV and SAV) and bacterial (*Y*. *ruckeri*) pathogens for both the stock concentration and the amount captured by the filters with the “sandwich” filtration method for reproducibility purposes.

### Validation of the “sandwich” filtration method using copy number estimation

As a final validation, two tests of the “sandwich” filtration method were set up for freshwater and artificial seawater to estimate pathogen copy numbers (S1 Table). For both experiments, 12.5 L of water were spiked with ISAV (final concentration of 400 TCID50/mL in 12.5 L), SAV3 (final concentration of 800 TCID50/mL in 12.5 L), and *Y*. *ruckeri* (initial OD600 = 2.241 in 1 mL added to 12.5 L). A volume of 1 L was filtered per sample using the EZ-Stream vacuum pump with a 3.8 to 4.0 L/min flow rate setting and 3 mL of DNA/RNA Shield™ were used as lysis buffer. Moreover, in these two tests, a 1:10 dilution in 1 mL of DNA/RNA Shield™ from the initial concentration vials for these pathogens was used to estimate the copy numbers/μL for quantification purposes of the stock vials. The recovery rate (%) of both filters after the filtration of 1 L of spiked water was calculated based on the formula [21, 52]:

$$Recovery\;(\%)=\frac{estimated\;copy\;numbers\;filtrated\;(GF+MCE)}{estimated\;copy\;numbers\;in\;spiked\;water}\;x\;100$$

### eDNA/eRNA extraction and qPCR / RT-qPCR assays

Automated DNA and viral RNA extractions were performed on a MagNA Pure 96 instrument (Roche) with the MagNA Pure 96 DNA and Viral NA Large Volume Kit (Roche, France), using Pathogen Universal LV protocol with a sample input volume of either 500 or 1000 μL and an elution volume of 50 or 100 μL, per sample respectively. RT-qPCR was carried out for ISAV [53], PRV1 [54] and SAV [24] with the Brilliant III Ultra-Fast QRT-PCR Master Mix (Agilent Technologies, USA) using the following protocol: 10 μL of 2× QRT-PCR master mix, 0.2 μL of 100 mM DTT, 1 μL of RT/RNase block, 1 μL of the 20× viral assay (6 μM of assay probe, 10 μM of each forward and reverse primers), and 5 μL extracted viral RNA. Thermocycling was performed on a CFX384 Bio-Rad and CFX-manager (software version 3.1, Bio-Rad, USA) under the following conditions: 10 min at 50°C, 3 min at 95°C, 45 cycles of 5 s each at 95°C, and 10 s at 60°C. qPCR was carried out for SGPV [31] using the Platinum™ Quantitative PCR SuperMix-UDG (Thermo Fisher Scientific, USA) with 5 μL of UDG platinum supermix, 0.9 μL of the viral assay (10 μM of each forward and reverse primers and probe), 0.3 μL MgCl_2_ (50 μM) and 2 μL extracted viral DNA. Thermocycling was performed on a CFX384 Bio-Rad and CFX-manager (software version 3.1, Bio-Rad, USA) under the following conditions: 2 min at 50°C, 15 min at 95°C, followed by 45 cycles of 15 s at 94°C, 30 s at 55°C and 15 s at 72°C.

qPCR was carried out for *Y*. *ruckeri* [48] with TaqMan™ Fast Advanced Master Mix (Applied Biosystems, USA) using the following protocol: 10 μL of 2× TaqMan Fast Advanced Master Mix, 5 μL of the bacterial assay (10 μM of assay probe and each forward and reverse primers), and 5 μL extracted DNA. Thermocycling was performed on a CFX384 Bio-Rad and CFX-manager (software version 3.1, Bio-Rad, USA) under the following conditions: 2 min at 50°C, 20 s at 95°C, 50 cycles of 3 seconds each at 95°C, and 20 seconds at 62°C.

All qPCR assays were analysed in duplicate, and no-template control (H_2_O) and water sample control from the MagNA Pure 96 extraction protocol were included on each plate as negative controls. Standard curves of serial dilution of the relevant target sequences (CFX-manager software version 3.1, Bio-Rad, USA) were used for the calculation of the concentration of use for the experiments. Primers and probes are shown in Table 1.

**Table 1:** Primer and probe sequences used for qPCR assays.

Pathogen	Oligonucleotides and probe
ISAV [53]	FW: 5’- CTACACAGCAGGATGCAGATGT -3’ REV: 5’- CAGGATGCCGGAAGTCGAT -3’ Probe: 5’-6-FAM- CATCGTCGCTGCAGTTC -MGB-NFQ-3’
PRV1 [54]	FW: 5’- TGCTAACACTCCAGGAGTCATTG -3’ REV: 5’- TGAATCCGCTGCAGATGAGTA -3’ Probe: 5’-6-FAM- CGCCGGTAGCTCT -MGB-NFQ-3’
SAV [24]	FW: 5’- CCGGCCCTGAACCAGTT -3’ REV: 5’- GTAGCCAAGTGGGAGAAAGCT -3’ Probe: 5’-6-FAM- CTGGCCACCACTTCGA -MGB-NFQ-3’
SGPV [31]	FW: 5’- ATCCAAAATACGGAACATAAGCAAT -3’ REV: 5’- CAACGACAAGGAGATCAACGC -3’ Probe: 5’-6-FAM - CTCAGAAACTTCAAAGGA -MGB-NFQ 3’
*Yersinia ruckeri—*CC 1 [48]	FW: 5’- GAATTAGGCGCAACTCAATTTGAC -3’ REV: 5’- ACTGGTAAGGGATGTTATGTTTCA -3’ Probe: 5’-VIC- TATGACGACTGAGTGTTTAC -MGB-NFQ-3’

### Statistical analyses

Statistical analyses were performed in GraphPad Prism v9.3.1 for Windows. Statistical tests were performed as One-Way ANOVA in case of a Gaussian distribution of residuals (Shapiro Wilk (W) test with p > 0.05) with the Tukey’s multiple comparisons test for multiple comparisons between means (* = p ≤ 0.05; ** = p ≤ 0.01; *** = p ≤ 0.001; **** = p ≤ 0.0001; n = 3). In the case of a non-Gaussian distribution of residuals, the non-parametric Kruskal-Wallis one-way analysis of variance test was applied with Dunn’s multiple comparisons test for multiple comparisons between means (* = p ≤ 0.05; ** = p ≤ 0.01; *** = p ≤ 0.001; **** = p ≤ 0.0001; n = 3).

## Results

### Freshwater bucket experiments

**Fig 3:**
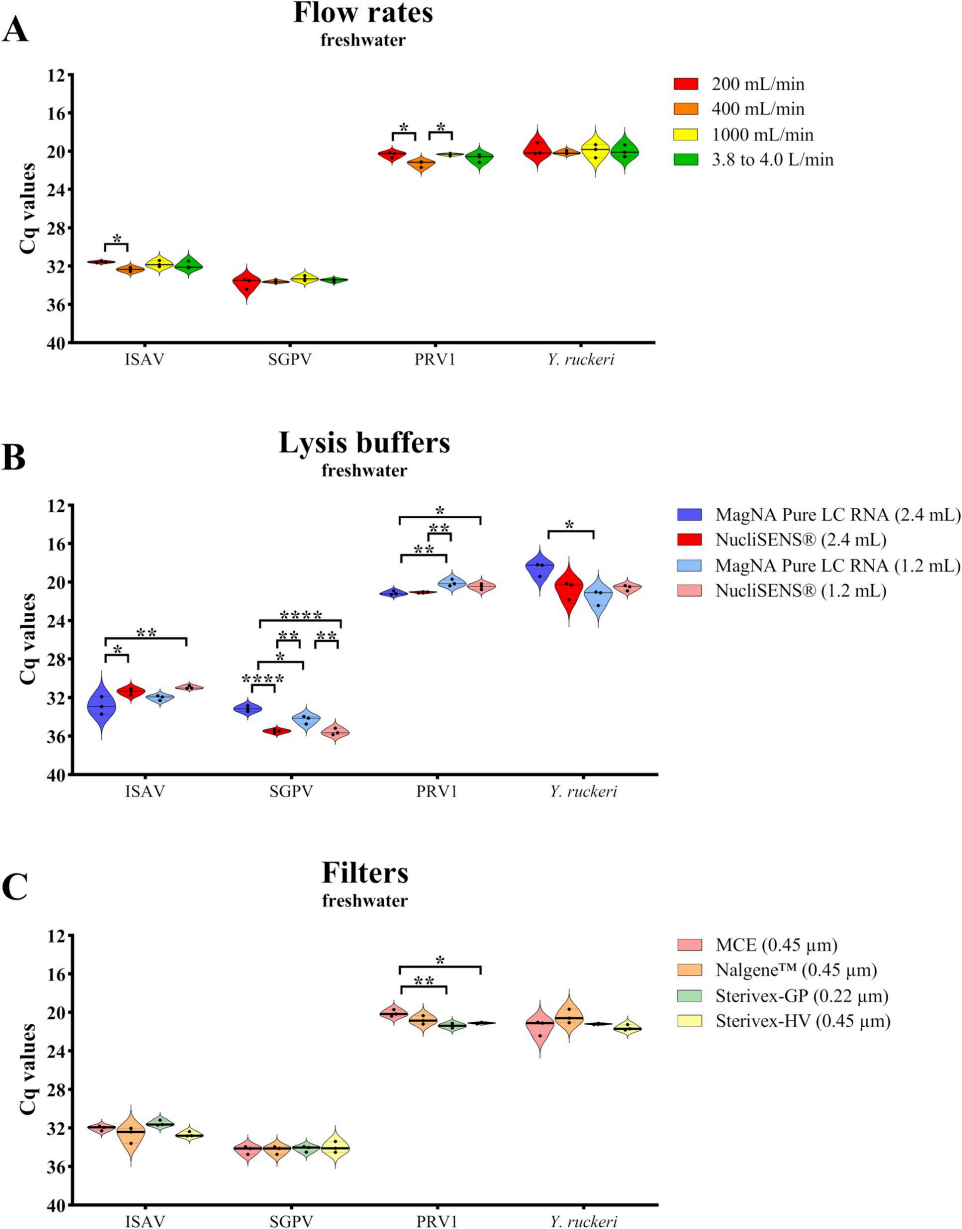
Experiment 1, trials A, B, C. Differences in DNA and RNA yield from qPCR and RT-qPCR analyses, respectively, for the four different fish pathogens ISAV, SGPV, PRV1 and *Y*. *ruckeri* in the freshwater bucket experiment. RNA or DNA yields are shown in terms of cycle quantification (Cq) values, where the y-axis is reversed from high to low numbers since RNA or DNA yield increases with decreased Cq-values. Violin plots show Cq median (solid line). Statistical tests were performed with the GraphPad Prism v9.3.1 (* = p ≤ 0.05; ** = p ≤ 0.01; **** = p ≤ 0.0001; n = 3). **A)** Trial A: test of four different filtration flow rates; 200, 400 and 1000 mL/min (peristaltic pump) and 3.8 to 4.0 L/min (vacuum pump), using MCE filter and 1.2 mL of MagNA Pure LC RNA lysis buffer. **B)** Trial B: test of two different lysis buffers (MagNA Pure LC RNA lysis buffer and NucliSENS® lysis buffer) in two different volumes (1.2 and 2.4 mL) using an MCE filter and 200 mL/min flow rate setting. **C)** Trial C: test of four different filters (MCE, Nalgene™, Sterivex-GP and Sterivex-HV) using 200 mL/min flow rate setting and 1.2 mL of MagNA Pure LC RNA lysis buffer.

In experiment 1, we aimed to improve earlier established methods by testing different filtration flow rates (trial A), lysis buffers (trial B) and filters (trial C) from a bucket of 50 L freshwater spiked with ISAV, SGPV, PRV1 and *Y*. *ruckeri* (Fig 3). In trial A, the four screened pathogens showed overall no significant differences in Cq values with the increase of the flow rate settings during the filtration of pathogens (Fig 3A). Only for ISAV and PRV1, we observed that a flow rate of 400 mL/min gave a higher Cq value (lower virus yield) in comparison to the other flow rates of the peristaltic pump and the vacuum pump (Fig 3A) with a statistical significance between 200 and 400 mL/min for ISAV and PRV1 (* = p ≤ 0.05) and between 400 and 1000 mL/min for PRV1 (* = p ≤ 0.05). However, the highest flow rate gave no significant changes in the virus quantification with qPCR assays compared to the lowest flow rate.

For trial B, two different lysis buffers for the filters were tested with two different volumes (1.2 and 2.4 mL). For the MagNA Pure LC RNA lysis buffer that is compatible with an automated RNA or DNA extraction, we found a significant difference in the Cq values between the two tested buffer volumes for the same pathogen (Fig 3B). We observed a lower Cq value (higher DNA yield) with 2.4 mL lysis buffer for SGPV and *Y*. *ruckeri* (* = p ≤ 0.05) compared to 1.2 mL (Fig 3B). In contrast, a higher Cq value (lower virus RNA yield) was achieved with 2.4 mL lysis buffer for PRV1 (** = p ≤ 0.01) (Fig 3B). The NucliSENS® lysis buffer that has been commonly used for ISAV and SAV [21, 22] gave significantly lower Cq values (higher RNA yield) for the 1.2 mL volume in comparison to 2.4 mL of the MagNA Pure LC RNA lysis buffer for ISAV (** = p ≤ 0.01) and PRV1 (* = p ≤ 0.05), but 1.2 mL volume of MagNA Pure LC RNA lysis buffer gave equal results as NucliSENS lysis buffer for PRV1. For SGPV, MagNA Pure LC RNA lysis buffer performed best for both volumes compared to NucliSENS® lysis buffer (** = p ≤ 0.01 for 1.2 mL and **** = p ≤ 0.0001 for 2.4 mL) (Fig 3B).

Trial C aimed to test four different filters (MCE, Nalgene™, the Sterivex-GP and the Sterivex-HV) and the results showed no significant differences for ISAV, SGPV and *Y*. *ruckeri* for all filter types while a significant difference was shown for PRV1 with lower Cq values (higher RNA yield) for the MCE filter compared to both Sterivex filters (* = p ≤ 0.05 with the Sterivex-HV; ** = p ≤ 0.01 with the Sterivex-GP) (Fig 3C).

**Fig 4:**
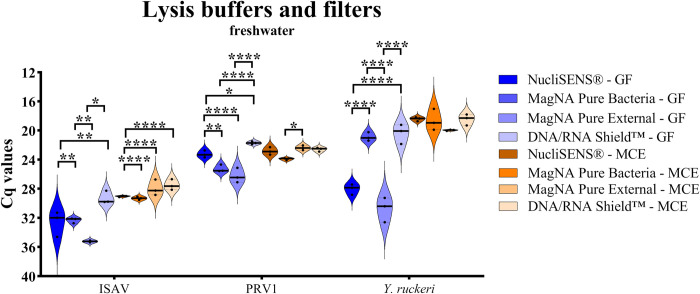
Experiment 2, trial D. Differences in DNA and RNA yield from qPCR and RT-qPCR analyses, respectively, for the three different fish pathogenic agents ISAV, PRV1 and *Y*. *ruckeri* in the second freshwater bucket experiment. RNA or DNA yields are shown in terms of cycle quantification (Cq) values, where the y-axis is reversed from high to low numbers since RNA or DNA yield increases with decreased Cq-values. Trial D tested four different lysis buffers in 4 mL volume (NucliSENS®, MagNA Pure 96 Bacteria, MagNA Pure 96 External, and DNA/RNA Shield™) using 200 mL/min flow rate setting (peristaltic pump) and “sandwich” filtration. The two different filters in the “sandwich” filtration (GF and MCE filters) were analysed separately. Violin plots show Cq median (solid line). Statistical significance levels were calculated only between different lysis buffers within the same filter type with the GraphPad Prism v9.3.1 (* = p ≤ 0.05; ** = p ≤ 0.01; **** = p ≤ 0.0001; n = 3).

The freshwater bucket, experiment 2, aimed to test if a “sandwich” filtration method would allow the filtration of larger water volumes without compromising the sensitivity for the concentration of viral RNA/DNA, and additionally test a wider range of temperature tolerant lysis buffers (NucliSENS®, MagNA Pure 96 Bacteria, MagNA Pure 96 External, and DNA/RNA Shield™). Statistical tests were performed only among different lysis buffers within the same type of filter. The GF and MCE filters were analysed separately but represented pairwise one filtration event. For the GF filter, the MagNA Pure 96 External lysis buffer performed worst of all tested buffers and gave the highest Cq values (lowest RNA/DNA yield) for all three pathogens (Fig 4). In contrast, DNA/RNA Shield™ showed significantly lower Cq values (higher RNA/DNA yield) for all three pathogens tested (Fig 4). For the MCE filter, the results were in general better and even higher than for GF filters. In addition, here, DNA/RNA Shield™ performed in general equally well or better than the other buffers (Fig 4). Specifically for the MCE filter, the MagNA Pure Bacteria lysis buffer showed significantly higher Cq values (lower RNA yield) for the two viruses than the other lysis buffers, while no statistical significance was shown for *Y*. *ruckeri* (Fig 4). The NucliSENS® lysis buffer, which showed lower Cq values (higher RNA yield) in the first buffer test analysis for ISAV (Fig 3B), showed significantly higher Cq values (lower RNA yield) than the DNA/RNA Shield™ (** = p ≤ 0.01 for GF; **** = p ≤ 0.0001 for MCE). The DNA/RNA Shield™ lysis buffer and the MagNA Pure External lysis buffer gave overall similar results for the viruses with the MCE filter, but for the bacterium, the DNA/RNA Shield™ lysis buffer gave a slightly lower although not significant Cq value. With the” sandwich” filtration method an increased amount of water could be filtered in the field (ca. 15%) before filter clogging compared to the use of MCE filter only (data not shown).

### Seawater bucket experiment

**Fig 5:**
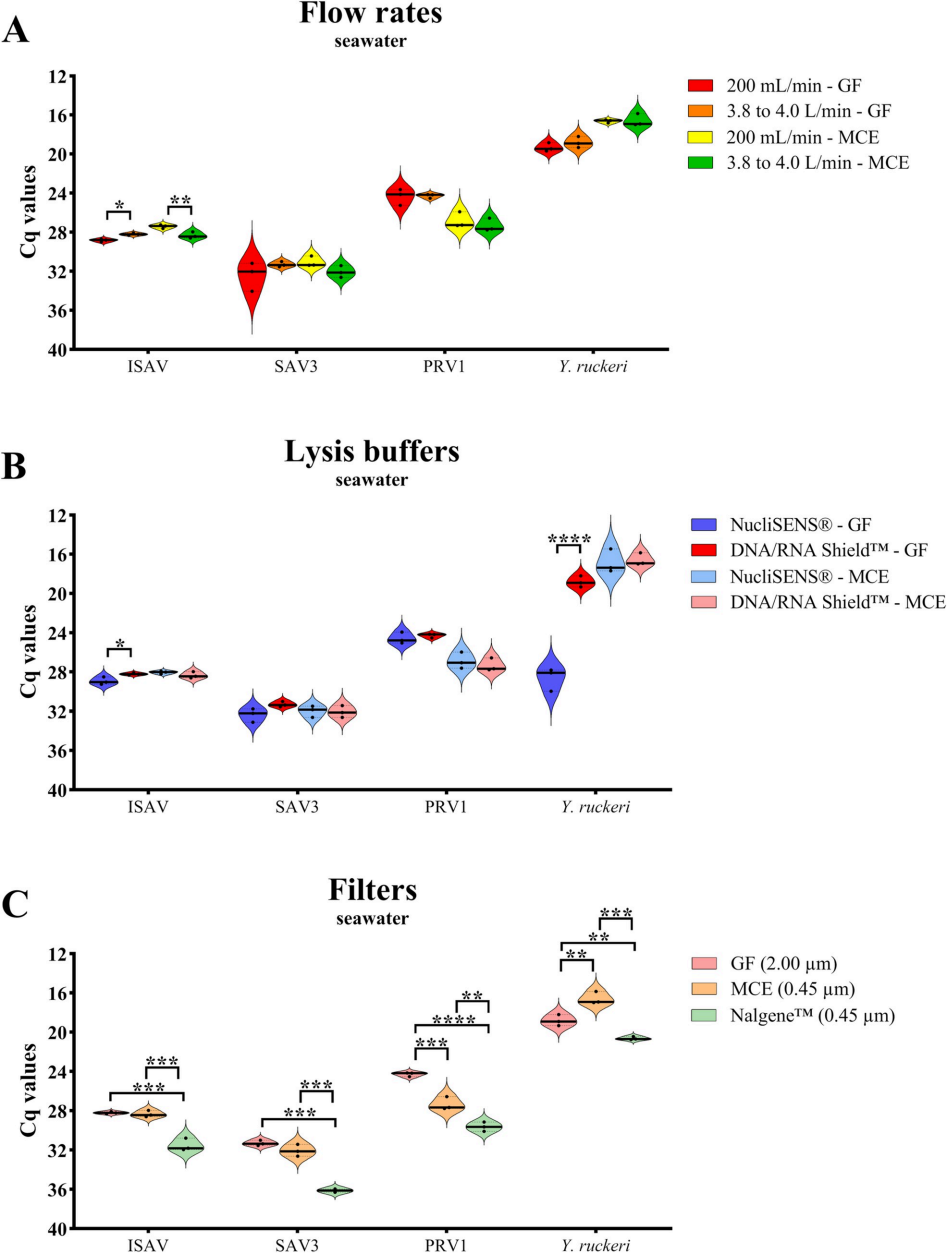
Experiment 3. Differences in DNA and RNA yield from qPCR and RT-qPCR analyses, respectively, for the four different fish pathogens ISAV, SAV3, PRV1 and *Y*. *ruckeri* in the seawater bucket experiment. RNA or DNA yields are shown in terms of cycle quantification (Cq) values, where the y-axis is reversed from high to low numbers since RNA or DNA yield increases with decreased Cq-values. Violin plots show Cq median (solid line). Statistical tests were performed with the GraphPad Prism v9.3.1 (* = p ≤ 0.05; ** = p ≤ 0.01; *** = p ≤ 0.001; **** = p ≤ 0.0001; n = 3). **A)** Trial E: Test of two different filtration flow rate settings, 200 mL/min (peristaltic pump) and 3.8 to 4.0 L/min (vacuum pump), using the “sandwich” filtration method and 3 mL of DNA/RNA Shield™ lysis buffer. **B)** Trial F: Test two different lysis buffers (3 mL of NucliSENS® and 3 mL of DNA/RNA Shield™) using the “sandwich” filtration method and the 3.8 to 4.0 L/min flow rate setting (vacuum pump). **C)** Trial G: Filter control test where the “sandwich” filtration method was compared to the Nalgene™ Single Use Analytical Filter Funnels. Trial G used 3 mL of DNA/RNA Shield™ with a flow rate setting of 3.8 to 4.0 L/min (vacuum pump).

In experiment 3, we aimed to test the most promising combinations of filters, buffers, and flow rates for freshwater in a seawater setup using artificial seawater spiked with ISAV, SAV3, PRV1 and *Y*. *ruckeri*. We tested: 1) the slowest and fastest filtration flow rate settings (trial E; 200 mL/min and 3.8 to 4.0 L/min); 2) two overall well-performing lysis buffers for all agents that also allowed storage at room temperature (trial F; NucliSENS® and DNA/RNA Shield™); and 3) a filter control (trial G) that tested one other and easy filtration method in addition to the “sandwich” filtration method. We detected significant differences between the two flow rates for each filter type only for ISAV in trial E (Fig 5A). The RNA yield was slightly higher (lower Cq value) at a higher flow rate for the GF filter (* = p ≤ 0.05), while the opposite was observed for the MCE filter (** = p ≤ 0.01) (Fig 5A). For the other pathogens, no significant differences between the two flow rates were shown (Fig 5A), similar to the freshwater bucket experiment results (Fig 3A). As the lysis buffer might influence the results based on its affinity to the type of pathogen, a similar analysis was performed with 3 mL of NucliSENS® lysis buffer based on promising results of this buffer with RNA viruses (Fig 3B). Here, no significant differences were shown between the high and low flow rates with one exception: For PRV1, the lowest Cq value (highest RNA yield) was gained with the low flow rate and GF filter (* = p ≤ 0.05) (S2 Fig).

In the lysis buffer test (trial F), the DNA/RNA Shield™ lysis buffer gave relatively stable results for both filter types, while NucliSENS® for some pathogens did not perform well with the GF filter (Fig 5B). We found a major difference in DNA yield in favour of the combination of GF filter and DNA/RNA Shield™ for *Y*. *ruckeri* (**** = p ≤ 0.0001), and a minor but still significantly lower Cq value (higher RNA yield) for ISAV for this filter and buffer combination (* = p ≤ 0.05) (Fig 5B).

Finally, for trial G (the filter control trial), the Nalgene™ filter showed significantly higher Cq values (lower DNA and RNA yield) than both filters in the “sandwich” filtration method for all screened pathogens (Fig 5C).

### Detection and quantification of ISAV, SAV3, and *Y*. *ruckeri* with the “sandwich” filtration method

In the two final validation experiments on freshwater and seawater, the 1:10 dilution from the stock concentration of ISAV, SAV3 and *Y*. *ruckeri* to estimate the corresponding Cq values and copy numbers are shown in Table 2. The filtered pathogen amount (Cq values) and corresponding copy numbers were estimated for both the freshwater (Table 3 and S2 Table) and seawater (Table 4 and S2 Table) validation experiment for each filter type. Table 5 (and S2 Table) shows the estimated copy numbers calculated from the initial concentration of use (stock) in 1L and the final recovery rate (GF+MCE filters) after the filtration of 1 L of spiked water of the two viruses for comparisons with previous publications.

**Table 2:** Stock concentrations (1:10) to estimate the corresponding Cq values and copy numbers. Values are presented as mean ± standard deviation from two technical replicates (n = 2).

	1:10 dilution in 1 mL DNA/RNA Shield™ from stock	Cq values	Estimated copy numbers/μL
ISAV	1 x 10^6^ TCID50/mL	23.24 ± 0.08	1.65 ± 0.08 x 10^4^
SAV3	1 x 10^7^ TCID50/mL	21.52 ± 0.77	4.92 ± 2.51 x 10^4^
*Y*. *ruckeri*	OD600 ≈ 2.241 in 1 mL	14.52 ± 0.06	4.88 ± 0.21 x 10^6^

**Table 3:** Freshwater experiment showing Cq values and estimated copy numbers after the filtration of 1 L of spiked water by the GF and MCE filters. 3.8 to 4.0 L/min flow rate setting and 3 mL of DNA/RNA Shield™ were used. Results are shown as mean ± standard deviation from six replicates (n = 6).

	GF—Cq values	GF—estimated copy numbers/μL	MCE—Cq values	MCE—estimated copy numbers/μL
ISAV	27.88 ± 0.71	6.39 ± 2.41 x 10^2^	28.49 ± 0.38	3.92 ± 1.07 x 10^2^
SAV3	28.27 ± 0.84	4.58 ± 1.74 x 10^2^	29.76 ± 0.41	1.50 ± 0.38 x 10^2^
*Y*. *ruckeri*	20.40 ± 0.19	1.04 ± 0.13 x 10^5^	20.00 ± 0.39	1.38 ± 0.35 x 10^5^

**Table 4:** Seawater experiment showing Cq values and estimated copy numbers after the filtration of 1 L of spiked water by the GF and MCE filters. 3.8 to 4.0 L/min flow rate setting and 3 mL of DNA/RNA Shield™ were used. Results are shown as mean ± standard deviation from six replicates (n = 6).

	GF—Cq values	GF—estimated copy numbers/μL	MCE—Cq values	MCE—estimated copy numbers/μL
ISAV	28.50 ± 0.73	4.27 ± 2.10 x 10^2^	27.72 ± 0.13	6.66 ± 0.06 x 10^2^
SAV3	27.79 ± 0.49	6.07 ± 2.24 x 10^2^	27.44 ± 0.11	7.34 ± 0.61 x 10^2^
*Y*. *ruckeri*	21.47 ± 0.53	5.40 ± 1.58 x 10^4^	20.44 ± 2.14	1.54 ± 0.83 x 10^5^

**Table 5:** Estimated copy numbers related to concentration in 1L and the recovery rate (GF+MCE) after the filtration of 1 L of spiked water. Results are shown as mean ± standard deviation from six replicates (n = 6).

	Estimated copy numbers/μL	Recovery (%) from freshwater	Recovery (%) from seawater
ISAV	6.60 (≈ 400 TCID50/mL)	14.06 ± 3.06	14.90 ± 3.53
SAV3	3.94 (≈ 800 TCID50/mL)	13.89 ± 3.82	30.66 ± 4.59

**Fig 6:**
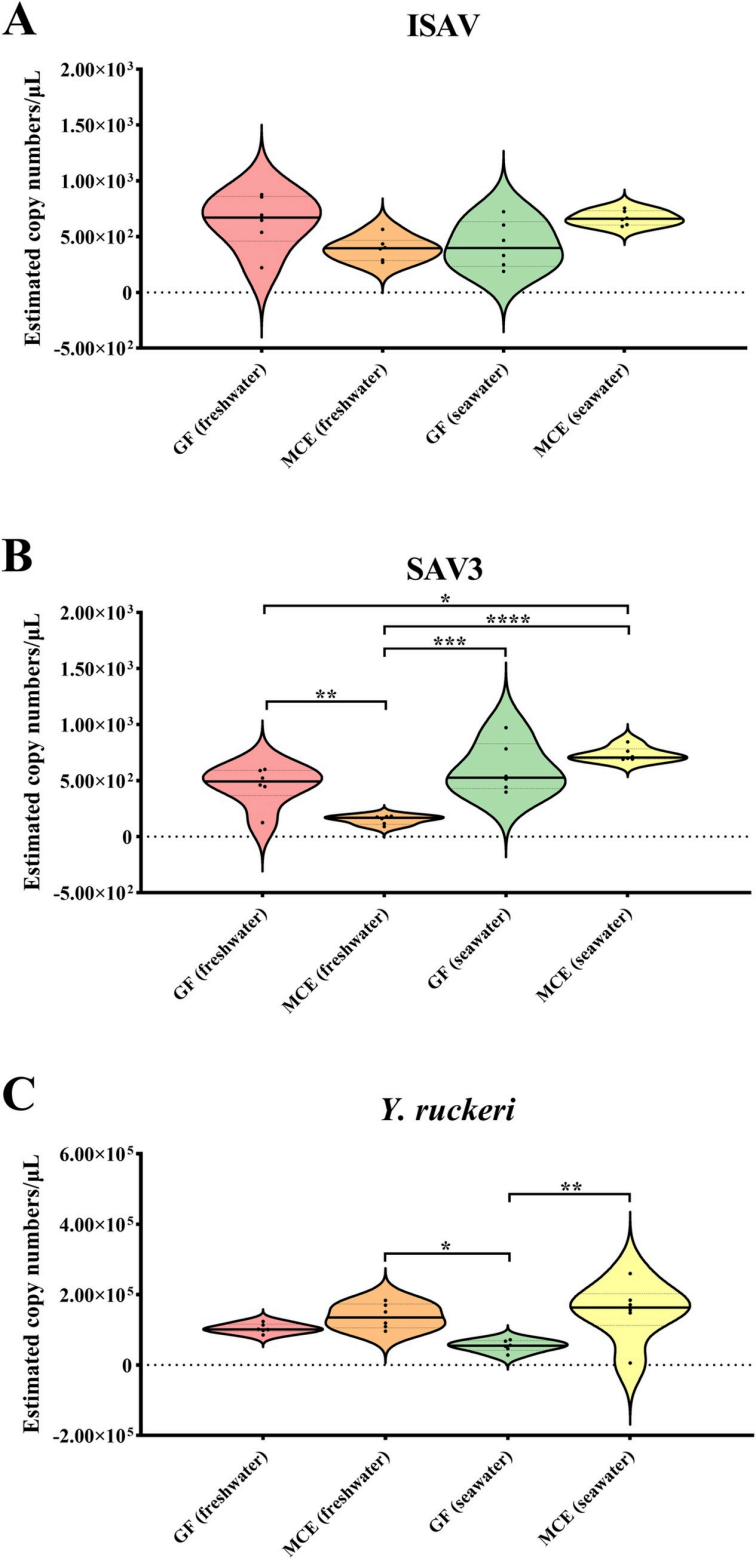
Estimated pathogen copy numbers after the filtration of 1 L of spiked water. **A)** ISAV **(**initial concentration 1 × 10^7^ TCID50/mL, final concentration of 400 TCID50/mL in 12.5 L**), B)** SAV3 **(**initial concentration 1 × 10^8^ TCID50/mL, final concentration of 800 TCID50/mL in 12.5 L) and **C)** Y. ruckeri **(**OD_600_ = 2.241 Abs in 1 mL). Filtration with the “sandwich” method was performed for both freshwater and seawater using a flow rate of 3.8 to 4.0 L/min and 3 mL DNA/RNA Shield™ lysis buffer. Violin plots with median (solid line). Statistical tests were performed with the GraphPad Prism v9.3.1 (* = p ≤ 0.05; ** = p ≤ 0.01; *** = p ≤ 0.001; **** = p ≤ 0.0001; n = 6).

The estimated copy numbers were also analysed for each pathogen to give a better representation of the differences between the two types of filters and the corresponding water environment (Fig 6). Significant differences were found only for SAV3 (Fig 6B) and *Y*. *ruckeri* (Fig 6C). SAV3 showed higher copy numbers in the seawater experiment with both filters reflecting its usual disease outbreaks as the disease occurs mostly during the salmonid seawater production phase (Fig 6B). *Y*. *ruckeri* showed a more heterogeneous estimation with the MCE filter in seawater compared to the GF filter, while no significant differences were found between the two types of filters in the freshwater experiment (Fig 6C). Overall, these last results have the purpose of giving guidelines to estimate the concentration in copy numbers of these three selected pathogens with a corresponding Cq value for reproducibility purposes and future screening baseline in the fish farms.

## Discussion

With the expansion of finfish aquaculture production, and the corresponding surge in infectious diseases challenging fish health and welfare, there is a pressing need to develop effective and less invasive monitoring approaches and proactive biosecurity measures to improve fish health and welfare, reduce the risk for spread of pathogens and mitigate environmental impact. The major challenges caused by infectious diseases in Norwegian aquaculture have also strongly contributed to a very high number of fish used in research experiments, calling for a stronger focus and implementation of the 3Rs in fish research [17]. A stronger incorporation of the 3R principles into surveillance and diagnostic practices in aquaculture would also be timely, and it would align with ethical and animal welfare considerations promoting more sustainable and cost-effective management practices. Therefore, monitoring of waterborne pathogens for surveillance purposes through filtration of water and subsequent molecular detection of target-specific DNA or RNA sequence motifs is an animal-friendly, non-invasive method with great potential for Atlantic salmon aquaculture and aligning with the 3R principles.

Current standards for diagnostics and surveillance of notifiable fish disease pathogens, for example, the surveillance of SAV infection in Norwegian fish farms, rely on traditional methods involving monthly sampling and analysis of fish from all marine farms with salmonids [2]. A recent study comparing eRNA detection of SAV from seawater with traditional surveillance methods concluded that SAV could be detected earlier from seawater samples than in fish samples in marine farm sites of Atlantic salmon that turned out SAV positive [8]. It also showed that the lower the amount of SAV virus particles detected in the water, the longer it took before SAV was detected in the fish samples. The authors, therefore, concluded that sampling of seawater every month for the surveillance of SAV has great potential as an alternative method for early detection of SAV in Atlantic salmon farms. In some SAV studies [8, 21, 23], water samples were taken in the field and then shipped within 24 h on ice to NVI for analysis. This method for filtration and capture of SAV from water poses a practical challenge in terms of method efficiency and robustness connected to the filtration workflow and dependency on cold temperature. Finally, the choice of isolation buffer with the highest recovery (NucliSENS® lysis buffer) [23] is partially dependent on cold storage conditions (up to 2 days storage at room temperature), which might be hard to achieve in seawater aquaculture facilities offshore. For practical purposes of early detection, this procedure slows down the analytical speed and it is not easy to harmonize with a broader eDNA/eRNA workflow that allows on-site water filtration and analyses of a broad range of prokaryotic and eukaryotic pathogens from one sample. We, therefore, focused on optimizing filtration flow rates (speed), filters and extraction buffers that would allow a smooth, relatively quick and temperature-independent surveillance of a broad range of fish pathogens in the freshwater and seawater aquaculture phases. In addition, we aimed at an initial sampling and filtering method that can be performed by farm personnel on-site to avoid shipment of water.

This study first demonstrated that relatively high flow rates (up to 4.0 L/min) had no significant impact on the pathogen quantification with qPCR compared to the low flow rates, which is a clear improvement in terms of time (and thus costs) needed for successful virus detection from water samples compared to previous recommendations [8, 21–23]. In addition, the actual achieved flow rate is dependent on several different factors, especially the water turbidity at each site, so methods not restricted to a specific optimal flow rate are more robust to handle in the farms. Moreover, the use of higher flow rates decreases the time spent on filtration of large water volumes during environmental sampling in the field. Our results also demonstrate that viruses are robust to several filtering approaches, allowing harmonized filtration workflow for multiple and biologically different pathogens of relevance to aquaculture.

Previous eDNA/eRNA studies present a wide variety of lysis buffer choices for pathogens, but often tailored specifically to each pathogen type, either connected to a taxonomic group or the nucleic acid target (DNA versus RNA). For instance, for pathogens that require DNA extraction, the specimens are typically preserved in ATL buffer (Qiagen) [14, 55], while NucliSENS® lysis buffer has been used for preservation and lysis of ISAV and SAV3 [8, 21–23]. Given the diverse range of pathogens present, simultaneous nucleic acid extraction for detection of multiple pathogens is very important for enhancing aquaculture biosecurity, and as a tool for fish health water-based epidemiology surveillance for potential pathogen transmission. In addition, the opportunity for storage and shipment in cold conditions can be limited at various field locations, which can negatively influence the stability of the samples and hamper the detection of pathogens. Therefore, in this study, we evaluated various lysis buffers to identify buffers that would allow temperature-independent storage and extraction of a wide range of pathogens and total nucleic acid extraction (DNA & RNA) without jeopardizing nucleic acid yield, but also buffers that would allow automated extraction. Five different lysis buffers were tested: the MagNA Pure LC RNA lysis buffer, the MagNA Pure 96 Bacteria lysis buffer and the MagNA Pure 96 External lysis buffer specific for nucleic acid isolation with the MagNA Pure 96 machine, the NucliSENS® lysis buffer previously used for viruses [8, 21–23] and the DNA/RNA Shield™, which is a DNA and RNA transport and storage medium for biological samples. Even if the choice of lysis buffer might be dependent on the type of pathogen relevant for screening, our results from the freshwater trials showed that the NucliSENS® lysis buffer might be a good choice if an RNA virus is the target, but this buffer allows up to 2 days storage at room temperature (protocols description of bioMérieux SA). In contrast, the MagNA Pure LC RNA specific for RNA isolation did not show lower Cq values (higher RNA yield) for any of the viruses used in this study and no statistical significance between the two buffers was shown for the bacterium. Moreover, the MagNA Pure LC RNA lysis buffer needs to be stored at 4°C both before and after sampling being less practical for use in the field. In contrast, DNA/RNA Shield™ gave the overall best results for all pathogens compared to the other lysis buffers both in freshwater and seawater experiments. This buffer may therefore represent a good candidate to preserve eDNA/eRNA filter samples in the field as it is stable at ambient temperatures for up to 30 days and completely inactivates infectious agents (viruses, bacteria, fungi, and parasites). Moreover, nucleic acid from samples stored in DNA/RNA Shield™ can be isolated directly without precipitation or reagent removal and it is compatible with most DNA and RNA purification kits, including the automated MagNA Pure systems.

The choice of filter is often under debate and many attempts to identify the golden standard have not succeeded although the Sterivex-HV filter (0.45 μm) has been sometimes used in eDNA inventory and surveillance studies [56–58]. However, when dealing with rare targets and the imperative of early detection, the restricted volume that can be filtered in turbid waters using a Sterivex filter presents limitations: its filtration workflow is time-consuming in terms of low flow rate and the choice of equipment requires disinfection between each sample (i.e., inlet tube to the filter). For fish viruses in water, previous studies showed a higher yield with the MCE filter [8, 21–23]. In our study, no significant differences were detected for the bacterium with this filter suggesting that the MCE filter may be also suitable for the detection and quantification of bacteria (eDNA) in water samples as previously shown [59]. Therefore, our findings bring out that the “sandwich” filtration method as a viable option for detecting and quantifying various types of pathogens in water samples. GF filter has been used for large water volume filtration and eukaryotic eDNA capture in the field [12–14] due to its relatively large pore size and allows the filtration of larger water volumes—often 5 to 10 L, which is used in the surveillance of crayfish plague and *G*. *salaris* [4, 7, 12–14]. Therefore, we designed a “sandwich” filtration method with an MCE filter on the bottom and GF filter on the top that aimed to capture the increasingly finer particulates at each stage providing filtration of all sizes of suspended solids based on results from previous studies [8, 9, 21–23, 51]. The recovery rate (%) of the “sandwich” filtration method was estimated for comparison with published results [21, 52] only for the two cultivable viruses (ISAV and SAV3). Our results showed a recovery rate of ca. 14% of ISAV in both freshwater and seawater while SAV3 showed a higher recovery in seawater (ca. 30%) compared to freshwater (ca. 14%) highlighting that the double filters do not increase the recovery rate but they are rather more successful in potentially allowing the filtration of a larger volume of water and detect pathogens present at low levels in the water compared to previously published results [21, 52]. Moreover, the difference in recovery rate in the two different environments between ISAV and SAV might be explained by the preferred disease outbreaks of SAV during the seawater production phase [21]. Although we were not able to estimate a recovery rate for PRV1, SGPV and *Y*. *ruckeri* as we lacked initial concentrations, our results, based on the Cq values, indicate that the protocol also produces comparable results to the ones obtained for ISAV and SAV. Similarly, gBlocks^TM^ copy numbers were estimated only for the pathogen with a known starting value (TCID50/mL or OD_600_) to be able to compare the copy numbers/μL before and after filtration. In addition, our results showed that the “sandwich” method decreased issues with clogging compared to the use of the MCE filter alone for water samples from the field.

In conclusion, the different methodologies tested in this study present a refinement to pathogen filtration workflow, storage and extraction under *in vitro* controlled conditions as promising up-and-coming tools for preventive biosecurity, which could potentially allow a less invasive monitoring approach for potential pathogen horizontal transmission via contaminated water (3Rs approach). Moreover, the “sandwich” filtration method has the additional benefit that can be easily applied in farms with no need for additional costly and time-consuming sterilization procedures or direct shipping of fish samples to laboratories for screening purposes.

## Supporting information

S1 Fig gBlock™ fragment with annotations of the target sequences. SAV = purple, Y. ruckeri = yellow, PRV1 = green, SGPV = brown and ISAV = blue. Annotation of the respective primers and probes: forward primers = dark green, reverse primers = light green and probes = red.
(TIF)

S2 Fig Experiment 3 –trial E. Differences DNA and RNA yield from qPCR and RT-qPCR analyses, respectively, for the four different fish pathogenic agents ISAV, SAV3, PRV1 and *Y*. *ruckeri* in the seawater bucket experiment. RNA or DNA yields are shown in terms of cycle quantification (Cq) values, where the y-axis is reversed from high to low numbers since RNA or DNA yield increases with decreased Cq-values. Trial E tested two different filtration flow rates: 200 mL/min (peristaltic pump) and 3.8 to 4.0 L/min (vacuum pump). Trial E used the “sandwich” filtration method and 3 mL of NucliSENS® lysis buffer. The two different filters in the “sandwich” filtration (GF and MCE filters) were analysed separately. Violin plots show Cq median (solid line). Statistical significance levels were calculated with the GraphPad Prism v9.3.1 (* = p ≤ 0.05; n = 3).
(TIF)

S1 Table Overview of bucket experiments 1–2 (freshwater), experiment 3 (seawater) and validation experiments (freshwater and seawater) with specified test parameters for each trial. (XLSX)

S2 Table gBlocks^TM^ calculations of copy numbers/μL and recovery (%). (XLSX)
