# Supplementary figures and images for: A refinement to eRNA and eDNA-based detection methods for reliable and cost-efficient screening of pathogens in Atlantic salmon aquaculture
Source: PLoS One. 2024 Oct 21;19(10):e0312337. doi: 10.1371/journal.pone.0312337 (PMC11493300; doi:10.1371/journal.pone.0312337)

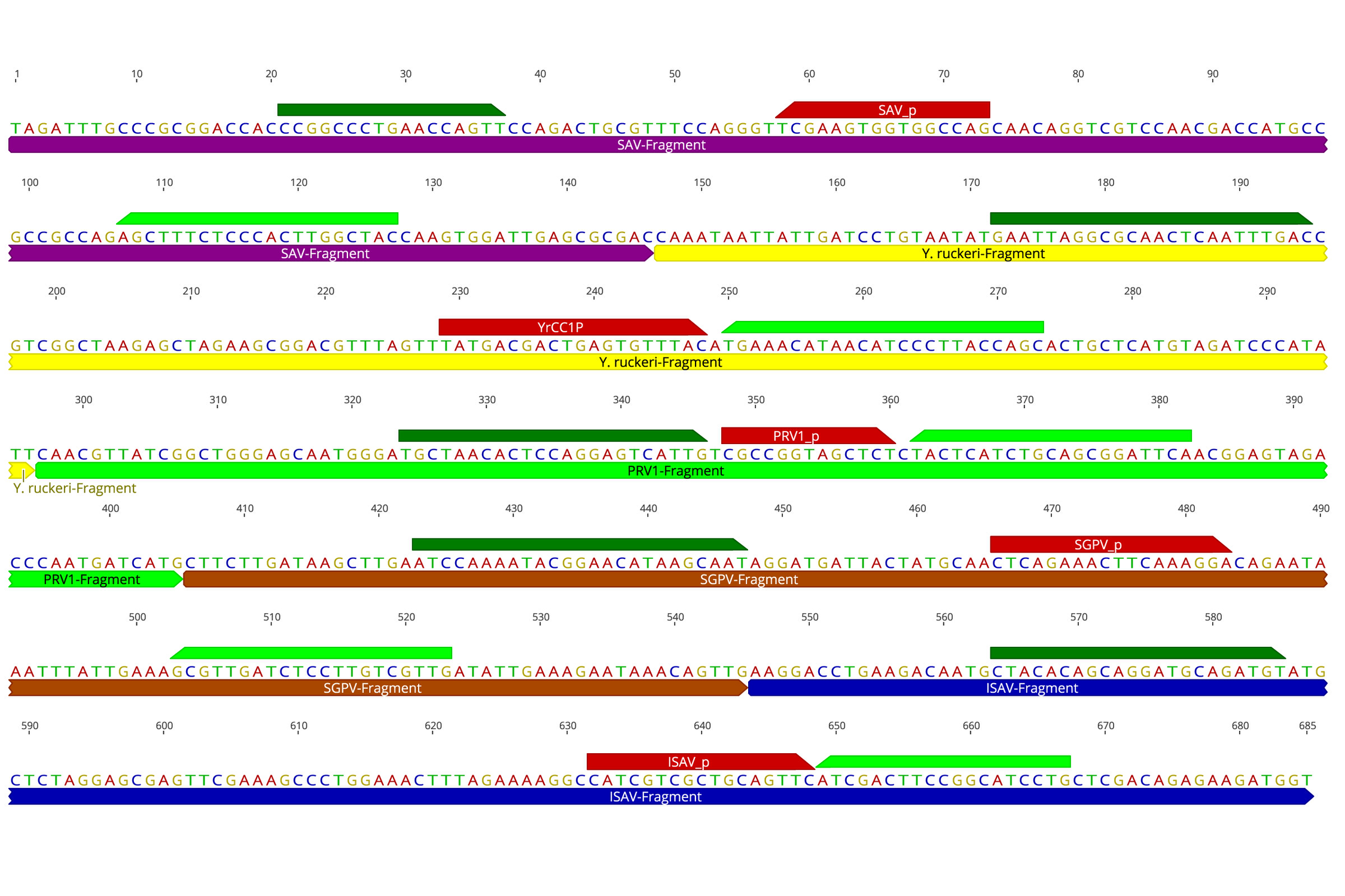

Supplement: S1 Fig — SAV = purple, Y. ruckeri = yellow, PRV1 = green, SGPV = brown and ISAV = blue. Annotation of the respective primers and probes: forward primers = dark green, reverse primers = light green and probes = red. (TIF) [file pone.0312337.s001.tif]

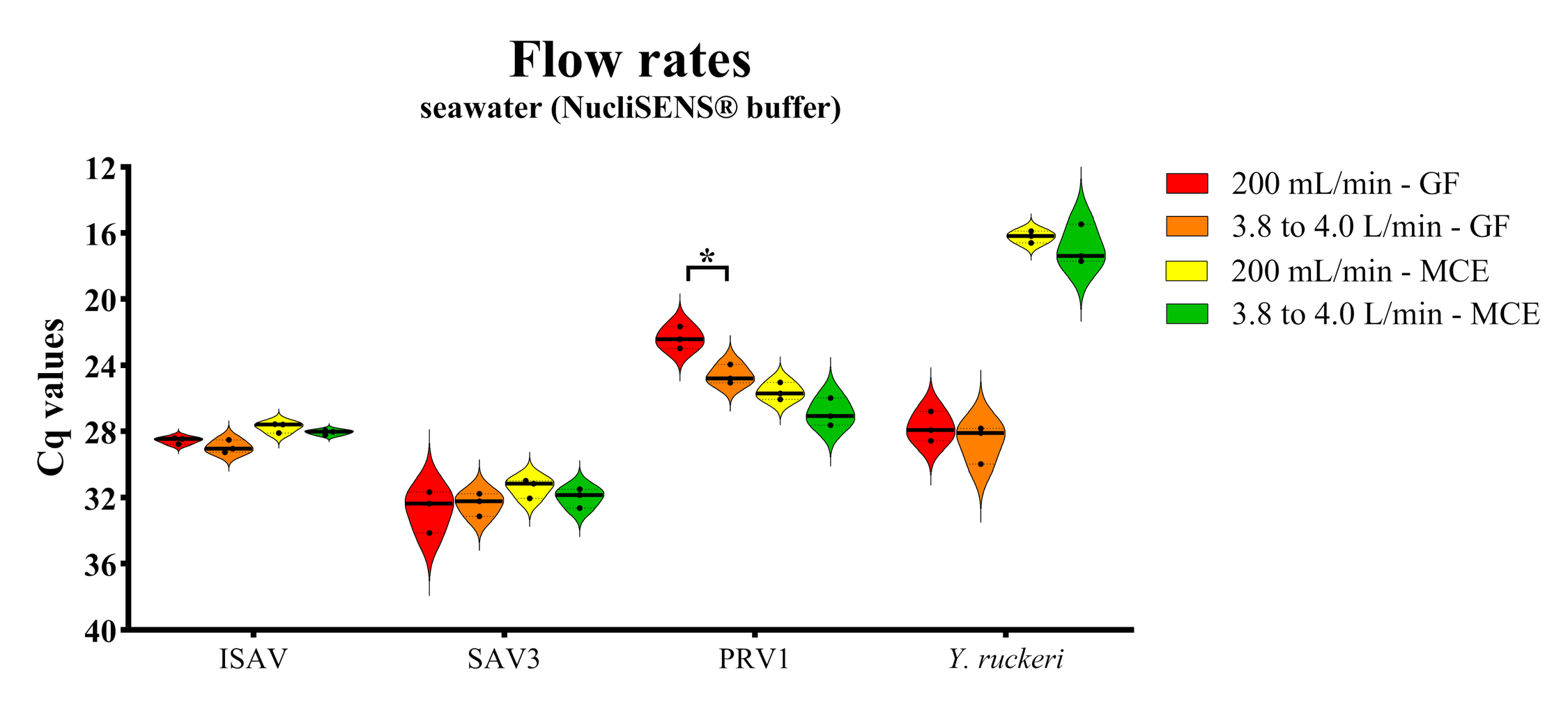

Supplement: S2 Fig — Differences DNA and RNA yield from qPCR and RT-qPCR analyses, respectively, for the four different fish pathogenic agents ISAV, SAV3, PRV1 and Y. ruckeri in the seawater bucket experiment. RNA or DNA yields are shown in terms of cycle quantification (Cq) values, where the y-axis is reversed from high to low numbers since RNA or DNA yield increases with decreased Cq-values. Trial E tested two different filtration flow rates: 200 mL/min (peristaltic pump) and 3.8 to 4.0 L/min (vacuum pump). Trial E used the “sandwich” filtration method and 3 mL of NucliSENS® lysis buffer. The two different filters in the “sandwich” filtration (GF and MCE filters) were analysed separately. Violin plots show Cq median (solid line). Statistical significance levels were calculated with the GraphPad Prism v9.3.1 (* = p ≤ 0.05; n = 3). (TIF) [file pone.0312337.s002.tif]
